# Case of Hare-Lip

**Published:** 1854-01

**Authors:** George Yates

**Affiliations:** Resident Surgeon of Queen's Hospital, Birmingham.


### ARTICLE XVII.

*Case of Hare-lip.* Reported by George Yates, Esq., Resident Surgeon of Queen's Hospital, Birmingham.

[This case was complicated, by a portion of bone projecting between the edges of the cleft, in which two teeth were placed, the whole being covered over by a cherry-like protuberance of integument. As this obstruction interfered with the proper adjustment of the edges of the wound, it was removed with a strong pair of forceps. The pins were removed on the fourth or fifth day, and fifteen days after the operation the patient was discharged; the wound having quite healed, and little or no scar left. Mr. Yates makes the following remarks:]

Hare-lip may or may not be combined with fissure of the palate, but the two are very frequently found together. The affection always occurs in the upper lip, and it assumes various and frequently even fantastic shapes. The cleft is sometimes simple; frequently, as in Green's case, there is a middle detached portion, and now and then two or more of such prominences. The cause of the disease is not obvious; it is most commonly attributed to an arrest of development; and is sometimes seen occurring in the same person or family, with malformations of the bladder, penis, feet and other parts. Sometimes the affection appears to be hereditary. Why these malformations occur we cannot say, and the answer generally given to the question—viz. that it is an arrest of development—is no answer at all, but only a statement of the fact itself.

Of course, there is but one mode of treatment to be adopted in these cases; but it is an important question, concerning which there is some difference of opinion, whether the operation should be performed while the child is young, or at what time of life it should be resorted to. Some would perform the operation before dentition commences. Lawrence "thinks it very desirable to remove the defect early on every account." Fergusson appears also to be an advocate for an early operation; but, on the other hand, Sir Astley and Samuel Cooper "recommends that the operation should not be undertaken till the child is about two years old, and has cut its teeth," because of "liability of young infants to sloughing, diarrhea, fever, and particularly the danger arising from hemorrhage and convulsions, which latter state is sometimes only to be checked by the removal of the threads, and allowing the original gap to be produced."

The propriety, however, of operation of the age of two years may well be questioned, as a child of that age is generally a very restless being, and not so likely to remain quiet as though only a few months old, nor nearly so readily to be confined and kept in order. Then, considering the dangers of operating on very young children, it would seem most proper that the operation should be put off for some years, or, at all events till such a time as the child may be able to understand the importance of remaining quiet, and aiding the surgeon as much as may be in the after-treatment of the case. The age, therefore, varies at which the operation may with most propriety be attempted, somewhat advanced age being perhaps preferable, next infancy, and lastly childhood. There is one circumstance, however, which must not be overlooked in coming to a determination regarding time, viz. that the fissure of the palate frequently closes of itself after the lip has been operated on during infancy.

The operation, however it may be performed, is intended to bring two surfaces previously ununited together, and for this, of course, an abrasion must be produced; but when there is a middle projecting portion, as happened in the case related, it becomes a question whether it should be taken away or left, and authorities are not agreed on this subject. Much, of course, must be left to the judgment of the operating surgeon, and it sometimes cannot be decided until the edges of the fissure are pared and approximated, whether it is proper to cut the projecting portion away or not. If the edges of the lip will come together nicely over the projecting portion, and without too much stretching, it may then be advisable to leave the part, especially as in some cases the contour of the face would be improved by such proceeding; but if it causes stretching of the parts, and projects more than is convenient, removal must be accomplished, care being taken not to cut away too much, lest falling in of the lip occur. Cooper says, "when the jaw itself projects, the common preliminary step to the operation consists in cutting away the bony prominence." Chelius also gives the same opinion. Fergusson, however, is adverse to removing the part totally; and Desault also objects to the proceeding, "the measure being seldom proper," in the opinion of this latter authority, who prefers "reducing the projection of the jaw by means of the pressure of a tight bandage," or, as in a case related in "Cooper's Dictionary," the projecting bones may be pushed backwards by means of a "spring truss, worn daily for several hours." Of course, these means are to be put in requisition some time before the actual operation; but they must be both tedious to the surgeon and irksome to the patient, and the writer cannot see that they are preferable to a slight use of the forceps or bone nippers. Gensoul, however, recommended the bending back of the projecting portion, and Mr. Haynes Walton has put the recommendation in practice with success, by partially dividing the projection, and then pressing it backwards.

*Med. Times and Gazette.*

